# Factors associated with women’s choice for mode of cervical sampling in future cervical cancer screening

**DOI:** 10.1371/journal.pone.0353180

**Published:** 2026-07-09

**Authors:** Natalija Berza, Jana Zodzika, Anda Kivite-Urtane, Alise Curkste, Ilva Pole, Mari Nygard, Kersti Parna, Anneli Uuskula

**Affiliations:** 1 Institute of Public Health, Riga Stradins University, Riga, Latvia; 2 Riga East University Hospital, Riga, Latvia; 3 Cancer Registry of Norway, Oslo, Norway; 4 Institute of Family Medicine and Public Health, University of Tartu, Tartu, Estonia; University of New South Wales, AUSTRALIA

## Abstract

**Background:**

Cervical cancer (CC), primarily caused by persistent high-risk human papillomavirus (HR-HPV) infection, remains a major public health challenge, with Latvia experiencing substantially higher incidence and mortality rates than the European average. HR-HPV self-sampling is an effective and acceptable screening method, yet some women continue to prefer clinician-collected sampling. Evidence on factors associated with reluctance to adopt self-sampling in the Baltic region is limited. This study examined demographic, socioeconomic, behavioral, and health-related characteristics linked to women’s preferences for future cervical cancer screening modalities.

**Materials and methods:**

This cross-sectional study was conducted in Latvia between February 2021 and April 2022. Women attending general practitioners or the Colposcopy Unit of Riga East University Hospital were invited to perform unsupervised HR-HPV self-sampling using written and visual instructions, followed by a structured questionnaire. Collected data included demographic and socioeconomic characteristics, health status, health behaviours, emotional responses to self-sampling, and preferred sampling modality for the next screening round. Descriptive statistics and Chi-square tests were used to assess differences in emotional responses and preferences. Factors associated with preferred sampling modality were identified through univariate and multivariate multinomial logistic regression. Statistical significance was set at p < 0.05.

**Results:**

A total of 1,217 women were included. Overall, 45.9% preferred clinician-collected cervical sampling, 42.6% preferred self-sampling, and 11.5% expressed no clear preference. Women who preferred self-sampling reported significantly lower embarrassment and discomfort during the procedure (p < 0.001). Most participants expressed positive emotional responses. The main reason for preferring clinician-collected sampling (81.0%) was a lack of confidence in performing self-sampling correctly. Multivariate analysis showed a higher preference for clinician-collection among women recruited from colposcopy settings and those with more recent gynecological visits, whereas former or occasional smokers were less likely to prefer clinician collection. Higher BMI (≥25) and non-Latvian nationality were associated with indecision regarding future sampling preference.

**Conclusion:**

HR-HPV self-sampling was generally well accepted and may help engage women less likely to attend traditional screening. Preference for clinician-collection was shaped mainly by medical history and nationality. Emotional factors may also influence screening choices and should be considered in future implementation strategies.

## Introduction

Cervical cancer (CC), primarily caused by high-risk human papillomavirus (HR-HPV), remains a major global health issue, particularly in low- and middle-income countries [1,2]. While the majority of HR-HPV infections resolve spontaneously, persistent infection is often linked to socioeconomic and behavioral factors, and it may promote progression to cervical intraepithelial neoplasia and invasive cancer [3,4].

Women who have never been screened or are under-screened face a significantly higher risk of invasive cervical cancer and are often difficult to reach through conventional methods [5,6]. Optimal screening coverage is a challenge that many countries face. In the UK, approximately 30% of women had not undergone screening in the last ten years. Meanwhile, Sweden saw a 10% rise in CC screening coverage after implementing HR-HPV self-sampling in April 2020 amidst the COVID-19 restrictions [7–9].

Latvia remains among the European countries with the highest cervical cancer incidence and mortality rates despite the introduction of an organized cervical cancer screening programme in 2009. Participation in screening has historically been low, and important implementation challenges persist, particularly among women living in rural areas, women with lower health literacy, and socioeconomically disadvantaged groups. In July 2022, Latvia initiated the transition from cytology-based screening to primary HR-HPV testing. Latvia also has a diverse ethnic composition, and previous studies have shown disparities in screening participation and healthcare access among minority populations. These factors highlight the need for acceptable and accessible screening approaches, including HR-HPV self-sampling, particularly in Latvia and the Baltic region as well [10,11–13,14].

HR-HPV self-sampling has become a promising approach to enhance participation in cervical cancer screening, effectively addressing significant practical and emotional obstacles [6]. The World Health Organization (WHO) advises that self-sampling should be made available as an alternative for women between the ages of 30 and 60, aiming for 70% screening coverage by the year 2030. [1].

Comprehensive worldwide evidence indicates that the accuracy of HR-HPV self-sampling is similar to that of samples collected by clinicians [15,16]. HR-HPV self-sampling could enhance screening participation since it is convenient for users, straightforward to integrate into screening initiatives, and efficiently utilizes medical resources, thus reducing the burden on healthcare systems [17].

Preference for clinician-collected cervical sampling has been associated with several factors, including older age, higher educational attainment, concerns about the reliability of self-sampling, cultural beliefs and norms, also, in some studies, higher body mass index (BMI) has been associated with this preference. Evidence also indicates that many patients value the opportunity for in-person interaction with a healthcare professional, which allows them to receive individualized counselling and to discuss additional health-related concerns [18–21].

Moreover, women’s choice of cervical cancer sampling modality is shaped not only by sociodemographic and practical considerations but also by the perceived acceptability of the procedure from the user’s perspective. In this context, acceptability is conceptualized broadly as women’s subjective experience of a sampling method, which may influence both the initiation and maintenance of its use. Although acceptability is a multidimensional construct, the present study concentrates specifically on feelings experienced during HR-HPV self-sampling, as these were directly assessed in the questionnaire and are particularly relevant for understanding preferences regarding future mode of cervical screening sampling and indirectly also participation [22].

Within this framework, negative feelings such as embarrassment and discomfort may function as perceived barriers, increasing the subjective burden associated with self-sampling. In contrast, confidence in one’s ability to carry out the procedure on their own reflects perceived self-efficacy and perceived behavioural control, both of which are central predictors of screening behaviour in established behavioural theories [23].

Findings from empirical studies on HR-HPV self-sampling consistently show that lower levels of embarrassment, enhanced privacy, and a greater sense of autonomy support the use of self-sampling. However, concerns about performing the test correctly and trusting the accuracy of the result continue to pose significant barriers for some women [24–26]. The objective of this study is to identify the determinants of preference for cervical screening sampling modality (self-sampling vs. clinician-collected) to ensure that HR-HPV self-sampling is universally adopted without creating new disparities in cervical cancer prevention.

## Materials and methods

### Setting

According to the 2022 IARC estimates, Latvia reported a high age-standardized incidence of cervical cancer (16.9 per 100,000 women), exceeding the European range of 15.7 in Eastern Europe to 6.4 in Northern Europe. Mortality was similarly elevated at 6.0 per 100,000, compared to 6.3–2.1 per 100,000 across Europe [10,11,27–29].

Several factors have been identified to explain Latvia’s elevated cervical cancer incidence, which is nearly double the EU average [27,11–13]. Although an organized screening program was introduced in 2009 for women aged 25–70, it relied on Leishman-stained cytology, a method with uncertain diagnostic validity until its replacement with liquid-based cytology in 2021. In 2022, the HR-HPV primary screening was introduced for women aged 30–70. Low participation has also been a challenge: screening uptake remained around 25% until 2016, increasing to 63.6% by 2024 [30]. Additionally, the limited involvement of general practitioners and insufficient program monitoring and quality control have further impeded effectiveness [10,11–13].

This study was conducted during the transitional period preceding nationwide implementation of HR-HPV primary screening in Latvia.

### Data source and study sample

A cross-sectional study was carried out between February 2021 and April 2022 in two distinct population groups: women aged 25–70 years from the general population and women referred for colposcopy following abnormal cytology results. Women from the general population were recruited from ten general practitioner (GP) practices located across all five regions of Latvia. These practices were chosen by convenience to secure broad geographical representation, and recruitment continued at each site until the predefined sample size was reached. GPs were engaged to participate in the study during professional conferences on cervical cancer prevention held in 2020. For the colposcopy group, women referred for colposcopy were enrolled consecutively at the colposcopy unit of Riga East University Hospital (REUH), the largest colposcopy center in Latvia.

Exclusion criteria included previous treatment for cervical precancerous lesions and age outside the 25–70 range. Sample size was determined based on the expected HR-HPV prevalence in both populations. Sample size calculations were based on expected HR-HPV prevalence in both populations, with detailed methodology reported previously [10,31–33].

Participants attending a healthcare facility (GP practice or colposcopy clinic) were invited to complete a paper-based questionnaire capturing demographic, socioeconomic, health-related, and behavioural information. Following questionnaire completion, women were provided with an HR-HPV self-sampling kit together with standardized written and illustrated instructions. Self-sampling was performed by the participants themselves in a healthcare setting immediately following the clinical visit, without direct assistance from healthcare staff during sample collection. Privacy was ensured during the procedure.

Following the completion of HR-HPV self-collection procedures, participants were requested to complete a procedure evaluation form that assessed participant emotional/psychological responses and preferred cervical sampling method for the next round of cervical cancer screening, aimed to capture their direct feedback and subjective experiences of the self-collection process. Sociodemographic, behavioural, and health-related variables were selected based on previously reported associations with cervical cancer screening participation and HPV self-sampling acceptability. Women’s monthly net income in the last year (EUR) was categorized using cut-off values derived from the quartiles of the median income distribution, ensuring statistically meaningful and population-relevant income groups.

### Ethics statements

#### Studies involving human subjects.

The study involved human participants and was reviewed and approved by the Riga Stradins University Ethics Committee (approval number: 6–1/07/33). All participants were informed about the purpose and procedures of the study and provided written informed consent prior to participation. Participation was voluntary, and respondents were assured of anonymity and confidentiality. Data were collected and analysed in accordance with applicable ethical standards and data protection regulations.

**Studies involving animal subjects**: Not applicable.

**Inclusion of identifiable human data**: No potentially identifiable human images or data are presented in this study.

### Preferred mode of future cervical cancer screening

As a primary outcome measure, participants were asked to indicate their preferred mode of cervical sampling for their subsequent screening round. Response options included “clinician-collected sampling,” “HR-HPV self-sampling,” and “didn’t give a preference,” allowing for the assessment of future screening intentions.

### Assessment of emotional responses during HR-HPV self-sampling

To assess participants’ emotional/psychological responses regarding HR-HPV self-sampling, women were asked how they felt after performing the procedure. They were presented with the following statements: “I felt embarrassed,” “I felt confident,” “I felt discomfort,” and “I felt intrigued/interested.” Items were adapted from previously published acceptability measures, as summarised in the systematic review by Nelson et al. (2017), which identified embarrassment, discomfort, and confidence as key emotional constructs influencing acceptability [24]. The items were translated and adapted to the local context to ensure comprehensibility and cultural relevance. Responses were recorded using a Likert-type scale reflecting the intensity of each emotional response. For each statement, participants indicated the intensity of their feeling using an ordered categorical scale with five options: “The feeling was not at all expressed,” “The feeling was rather not expressed,” “Neutral - the feeling was neither expressed nor not expressed,” “The feeling was rather expressed,” or “The feeling was very strongly expressed.” For statistical analysis, the first two and the last two categories were merged (i.e., “not at all/ rather not expressed” and “rather/ very strongly expressed”). An additional option, “Hard to say/don’t know,” was included for participants who could not provide a definitive response. Finally, participants were asked an open-ended question to explain their reasons for preferring clinician-collected sampling for future cervical cancer screening.

### Statistical analysis

Descriptive statistics, such as proportions for categorical variables, were performed to determine the preference of the HR-HPV self-sampling test estimate. They were also used to determine the prevalence of dependent variables across the strata of independent variables in Supplement 1.

The dependent variable is the preference for the mode of cervical sampling during the next round of CC screening. In Table 2, the four emotional/psychological responses are listed while undergoing the self-sampling procedure during the study. The Chi-square test was used to determine the statistical significance of the mentioned stratified prevalence differences.

Univariate and multivariate multinomial logistic regression were used to identify factors associated with preference for the mode of cervical sampling during the next round of CC screening. The adjusted model contains all the independent variables shown in Table 3. Before inserting the variables into the model, it was checked whether there was any collinearity between them, and none was found. Results were considered statistically significant at p < 0.05.

Data was processed using IBM SPSS Statistics (Statistical Package for the Social Sciences) Version 26.0. 95% Confidence Intervals (95% CI) were calculated for prevalence estimates using the OpenEpi calculator [34].

## Results

### Participant enrolment and refusal rates

A total of 1,413 (800 from general practitioners (GP) practices and 613 from the colposcopy unit) women were invited to participate in the study. The refusal rate (the proportion of women invited to participate in the study but who declined) was 11.1% in the general population group and 4.0% in the colposcopy group. The predominant reasons for refusal were consistent across both groups: 47.1% (n = 92) of participants cited reluctance to undergo self-sampling, 20.6% (n = 40) cited lack of time, and 14.7% (n = 29) asserted that the gynaecologist had already conducted a comprehensive examination, rendering the additional test unnecessary. Evaluation questions specifically on the emotional/psychological responses during the HR-HPV self-sampling procedure and preferred mode of sampling in the next screening round were completed, all in all, by 1,217 participants – 769 of these women were recruited in GP practices, while the remaining 448 were from the colposcopy unit. This makes our sample for this study.

### Participant demographics and socioeconomic characteristics

The study population predominantly consisted of women aged 25–49 years (77.2%), with the majority identifying as Latvian (74.9%). The majority were married or cohabiting (70.7%), and approximately half held a university-level education (54.3%), while 45.7% had primary or secondary education. In terms of income, 61.5% reported a personal monthly net income between 401 and 1,000 EUR, indicating a predominance of middle-income earners, with 21.9% reporting ≤400 EUR and 16.7% reporting >1,000 EUR. As shown in Supplement 1, several sociodemographic and health-related characteristics differed significantly across the three preference groups.

Non-Latvian women were more represented in the group preferring clinician-collected cervical sampling than in the self-sampling group (27.2% vs. 21.6%, *p* = 0.031). Similarly, women from the colposcopy study group were more strongly represented among those choosing clinician-collected sampling compared with women from the general population (44.1% vs. 30.8%, *p* < 0.001).

The distribution of last gynecologist visit categories differed significantly across the three preference groups (*p <* 0.001). In the clinician-collected sampling group, women who had visited a gynecologist within the past year were proportionally most highly represented (69.7%, *p <* 0.001) in comparison with less recent visits. In the self-sampling group, women whose last gynecologist visit occurred 1–3 years ago formed a comparatively larger proportion (25.2%, *p <* 0.001) than in the clinician-collected sampling group (19.7%, *p <* 0.001) or among undecided participants (17.9%, *p <* 0.001). Likewise, women who had not visited a gynecologist for more than three years constituted a greater proportion of the self-sampling group (8.7%, *p* < 0.001) compared with the clinician-collected sampling group (3.0%, *p <* 0.001). In contrast, women with a BMI (≥25) were more frequently represented among those who were undecided about their preferred screening method (60.9%) than among those preferring either clinician-collected (45.1% *p* < 0.001) or self-sampling (44.3%, *p* < 0.001). No significant differences between the preference groups were observed for age, education, income, marital status, smoking, alcohol use, number of sexual partners, or chronic disease status.

### Preferred mode of cervical cancer screening

Overall, 45.9% (95% CI: 43.1–48.7) indicated a preference for clinician-collected sampling, 42.6% (95% CI: 39.9–45.4) preferred self-sampling, and 11.5% (95% CI: 9.8–13.4) reported no clear preference.

### Participants’ emotional/psychological responses regarding HR-HPV self-sampling

**Fig 1:**
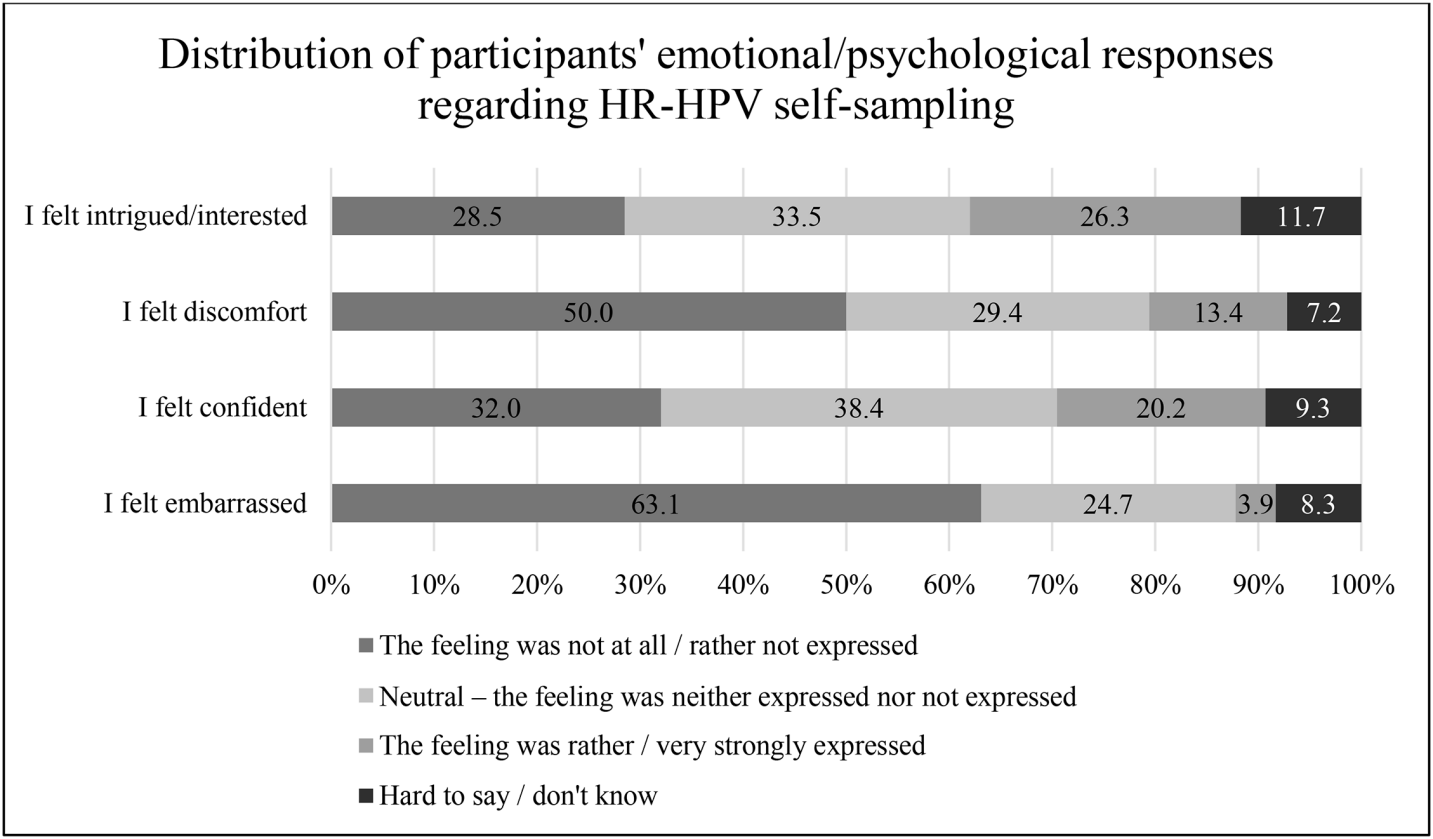
Proportion of women (%) as per their emotional/psychological responses about the HR-HPV self-sampling procedure.

As shown in Fig 1, the majority of participants did not express negative emotional/psychological responses, with 63.1% (95% CI: 60.1–66.1) reporting ‘’The feeling was not at all expressed/rather not expressed for embarrassment, and 50.0% (95% CI: 47.0–53.1) for discomfort. Conversely, positive emotional/psychological responses were more frequently reported, though with varying intensity. Specifically, 26.3% (95% CI: 23.7–29.1) of women stated ‘’The feeling was rather/ very strongly expressed” strongly expressed feeling “intrigued/interested,” and 20.2% (95% CI: 17.9–22.8) reported feeling “confident” about performing self-sampling. A considerable proportion of participants remained neutral across all emotional dimensions: 29.4% (95% CI: 26.6–32.2) for discomfort, 38.4% (95% CI: 35.5–41.4) for confidence, 24.7% (95% CI: 22.1-27.5) for embarrassment, and 33.5% (95% CI: 30.7-36.5) for intrigued/interested. Only a small percentage, ranging from 7.2% (95% CI: 5.7–8.9) to 11.7% (95% CI: 9.9–13.8) across different emotional/psychological responses, selected the answer ‘’Hard to say/don’t know,” indicating some uncertainty.

Table 1 presents a comparison of participants’ emotional/psychological responses to the HR-HPV self-sampling procedure according to their preferred mode of cervical sampling in the next round of screening. Statistically significant differences were observed for embarrassment (*p* < 0.001), discomfort (*p* < 0.001), and confidence (*p* = 0.017). Women preferring clinician-collected cervical sampling more frequently reported both embarrassment and discomfort as ‘rather/very strongly expressed’ compared with women preferring self-sampling (embarrassment 6.4% vs. 1.6%; discomfort 18.2% vs. 9.4%). These differences were significant in pairwise comparisons with both the ‘neutral’ and ‘not expressed’ categories, as indicated by the letter groupings in Table 1. Conversely, participants who favored self-sampling more frequently rated these emotional/psychological responses as “not expressed” or “neutral.” Regarding confidence, women who preferred self-sampling more often reported “the feeling was rather/very strongly expressed” (23.2%) compared with those favoring clinician-collected sampling (17.1%), indicating a greater comfort with the procedure. No statistically significant differences were observed for the feeling of interest or intrigue (*p* = 0.088), suggesting that curiosity and engagement toward the self-sampling procedure were comparable between the groups.

**Table 1:** Number and proportion (%) of responders according to emotional/psychological responses about self-sampling procedure, as per the preference of sampling method for the next round of CC screening.

Self-Reported Item	Sampling Preference	N	Not/Rather Not Expressed	Neutral	Rather/Very Strongly Expressed	Hard to Say/Don’t Know	p-value
**I felt embarrassed**	**HR-HPV self-sampling**	438	64.8% ^a^	27.4% ^b^	1.6% ^abc^	6.2% ^c^	<0.001***
	**Clinician-collected sampling**	435	60.9% ^a^	23.7% ^b^	6.4% ^abc^	9.0% ^c^	
**I felt confident**	**HR-HPV self-sampling**	465	29.7% ^a^	40.2% ^b^	23.2% ^ac^	6.9% ^bc^	0.017*
	**Clinician-collected sampling**	468	34.0% ^a^	38.0% ^b^	17.1% ^ac^	10.9% ^bc^	
**I felt discomfort**	**HR-HPV self-sampling**	447	55.9% ^abc^	29.3% ^ad^	9.4% ^bd^	5.4% ^c^	<0.001***
	**Clinician-collected sampling**	456	43.0% ^abc^	31.1% ^ad^	18.2% ^bd^	7.7% ^c^	
**I felt intrigued/interested**	**HR-HPV self-sampling**	454	28.4%	31.9%	30.6%	9.0%	0.088
	**Clinician-collected sampling**	452	27.0%	35.6%	24.8%	12.6%	

a, b, c, d – earmarks the pairs of stratified percentages where the p < 0.05

The most frequently reported reason for preferring clinician-collected cervical sampling was a lack of confidence in performing self-sampling correctly (81.0%). Other reasons included greater convenience and reassurance associated with the clinician’s involvement (14.6%), and less common reasons like discomfort with self-sampling (2.5%), habit or prior experience with clinician-collected sampling (0.8%), identifying herself as a high-risk patient (0.3%), and fear of causing self-injury during self-sampling (0.3%).

Using multivariate logistic regression analysis, several factors were found to be significantly associated with a preference for clinician-collected cervical sampling (Table 2). Women belonging to the colposcopy study group had higher odds of preferring clinician-collected testing compared with those from the general population (aOR = 1.84, 95% CI: 1.37–2.47). Women who had had a visit with a gynecologist in the last year were more likely to prefer clinician-collected sampling than those whose last visit to a gynecologist was less recent (aOR = 0.34, 95% CI: 0.18–0.64). Similarly, those who reported having quit or only occasionally smoked were less likely to prefer clinician-collected sampling compared with never-smokers (aOR = 0.64, 95% CI: 0.46–0.90). In the unadjusted model, non-Latvian women were more likely to prefer clinician-collected cervical sampling compared with Latvian participants; however, this association did not remain significant after adjustment for other variables. Other sociodemographic and health-related variables, including age, marital status, education, monthly income, alcohol use, number of sexual partners, BMI, and chronic disease status, were not significantly associated with testing preference in the adjusted model.

**Table 2:** Factors associated with preferring cervical sampling performed by a clinician in the next screening round (reference: self-sampling).

Variable	n	% of women preferring clinician-collected cervical sampling in the next round of screening	OR	95% CI	aOR*	95% CI
Age (years)**						
≤49	436	46.4	1.05	0.79-1.40	0.94	0.65-1.36
50+	122	43.9	1		1	
**Nationality**						
Non-Latvian	152	49.7	**1.36**	**1.03-1.80**	1.29	0.94-1.76
Latvian	406	44.6	1		1	
**Marital status**						
Divorced, widow	74	47.1	1.48	0.94-2.32	1.39	0.82-2.34
Married, cohabiting	408	47.4	1.39	0.99-1.94	1.34	0.92-1.94
Single	76	38.2	1		1	
**Education**						
Primary, secondary	245	44.1	0.93	0.73-1.18	0.89	0.67-1.20
University	313	47.4	1		1	
**Women’s monthly income neto in last year (EUR)**						
≤400	119	44.7	1.01	0.69-1.49	0.96	0.60-1.51
401-1000	347	46.4	1.06	0.77-1.48	1.11	0.77-1.59
1001+	92	45.3	1		1	
Smoking status***						
Daily	133	42.2	0.80	0.60-1.07	0.73	0.51-1.04
Sometimes, quit	120	42.6	**0.74**	**0.55-0.99**	**0.64**	**0.46-0.90**
Never	305	49.2	1		1	
**Alcohol use in last 12 months**						
Once per week or more often	30	39.0	0.83	0.49-1.40	1.07	0.60-1.91
Rarely	279	47.0	1.07	0.83-1.38	1.17	0.89-1.55
Never	213	44.7	1		1	
**Number of lifetime sex partners**						
4+	283	44.3	1.00	0.70-1.43	0.89	0.59-1.35
2-3	163	48.8	1.16	0.78-1.71	1.05	0.69-1.61
0-1	76	43.4	1		1	
**BMI**						
25+	250	44.6	1.03	0.81-1.32	1.08	0.82-1.43
<25	304	47.3	1		1	
**Chronic diseases**						
Yes	189	45.4	1.00	0.78-1.29	1.07	0.81-1.43
No	369	46.1	1		1	
**Last visit to gynaecologist**						
Don’t know, never	42	50.6	1.17	0.71-1.92	0.81	0.46-1.43
More than 3 years ago	17	21.0	**0.31**	**0.17-0.54**	**0.34**	**0.18-0.64**
1-3 years ago	110	41.4	**0.68**	**0.51-0.91**	0.75	0.54-1.04
Last year	389	49.4	1		1	
**Study group**						
Colposcopy	246	54.9	**1.77**	**1.38-2.27**	**1.84**	**1.37-2.47**
General population	312	40.6	1		1	

* aOR – adjusted odds ratio, adjusted for all factors in the table

** Age groups (<49 and ≥50 years) were defined based on prior evidence of lower screening rates among women ≥50 [26].

*** “Sometimes/ ex-smoker” refers to women with previous or occasional smoking exposure who did not report daily smoking. These subgroups were combined for analytical purposes.

Table 3 presents the factors associated with having no preference for the mode of cervical sampling in the next round of screening. In the adjusted model, women with higher BMI (≥25) were significantly more likely to report no clear preference compared with those with lower BMI (<25) (aOR = 2.13, 95% CI: 1.37–3.32). Non-Latvian nationality was also associated with a higher likelihood of expressing no preference (aOR = 1.64, 95% CI: 1.02–2.64). No significant associations were found for age, education, marital status, income, smoking, alcohol use, number of lifetime sex partners, chronic diseases, time since last gynecologist visit, or study group.

**Table 3:** Factors associated with having no preference (“hard to say”) regarding the mode of cervical sampling in the next screening round (reference: self-sampling).

Variable	n	% of women having no preference for the mode of cervical sampling in the next round	OR	95% CI	aOR*	95% CI
Age (years)**						
≤49	102	10.9	0.79	0.52-1.21	1.18	0.66-2.09
50+	38	13.7	1		1	
**Nationality**						
Non-Latvian	42	13.7	**1.56**	**1.03-2.37**	**1.64**	**1.02-2.64**
Latvian	98	10.8	1		1	
**Marital status**						
Divorced, widow	21	13.4	1.10	0.58-2.10	0.96	0.44-2.07
Married, cohabiting	90	10.5	0.80	0.50-1.29	0.71	0.41-1.23
Single	29	14.6	1		1	
**Education**						
Primary, secondary	74	13.3	1.33	0.92-1.94	1.18	0.74-1.89
University	66	10.0	1		1	
**Women’s monthly income neto in last year (EUR)**						
≤400	33	12.4	1.17	0.64-2.15	0.95	0.47-1.94
401-1000	85	11.4	1.09	0.64-1.84	0.91	0.51-1.63
1001+	22	10.8	1		1	
**Smoking status**						
Daily	46	14.6	1.30	0.85-2.00	1.25	0.74-2.14
Sometimes, quit	29	10.3	0.84	0.52-1.36	0.76	0.43-1.34
Never	65	10.5	1		1	
**Alcohol use in last 12 months**						
Once per week or more often	12	15.6	1.22	0.59-2.50	1.15	0.50-2.61
Rarely	63	10.6	0.89	0.59-1.33	0.89	0.57-1.39
Never	58	12.2	1		1	
**Number of lifetime sex partners**						
4+	72	11.3	0.84	0.49-1.43	0.83	0.44-1.55
2-3	30	9.0	0.70	0.38-1.30	0.74	0.38-1.42
0-1	23	13.1	1		1	
**BMI**						
25+	84	15.0	**1.96**	**1.33-1.87**	**2.13**	**1.37-3.32**
<25	54	8.4	1		1	
**Chronic diseases**						
Yes	51	12.3	1.12	0.76-1.65	0.95	0.61-1.50
No	89	11.1	1		1	
**Last visit to gynaecologist**						
Don’t know, never	12	14.5	1.55	0.76-3.16	1.03	0.45-2.31
More than 3 years ago	19	23.5	1.58	0.88-2.84	1.22	0.62-2.41
1-3 years ago	25	9.4	0.71	0.44-1.17	0.58	0.33-1.03
Last year	84	10.7	1		1	
**Study group**						
Colposcopy	42	9.4	0.96	0.64-1.44	0.90	0.55-1.47
General population	98	12.7	1		1	

* aOR – adjusted odds ratio, adjusted for all factors in the table

** Age groups (<49 and ≥50 years) were defined based on prior evidence of lower screening rates among women ≥50 [26].

*** “Sometimes/ ex-smoker” refers to women with previous or occasional smoking exposure who did not report daily smoking. These subgroups were combined for analytical purposes.

## Discussion

This study focuses on women’s preferences regarding different modes of cervical sampling, specifically comparing HR-HPV self-sampling with clinician-collected cervical sampling. This study assessed the emotional/psychological responses to the self-sampling procedure and preferred mode of sampling in the next round of screening among different groups of women, including those from the general population and those referred for colposcopy. To the best of our knowledge, this is the first study in the Baltic region to examine women’s feelings during the HR-HPV self-sampling procedure. These findings are particularly relevant in Latvia, where HR-HPV self-sampling has not yet been implemented within the organized screening programme.

The present study found that participants generally reported positive or neutral emotional/psychological responses during the HR-HPV self-sampling procedure, with few women expressing embarrassment or discomfort. These findings align with previous research indicating that self-sampling is usually perceived as a comfortable and acceptable procedure among women in different populations and especially among screening non-attenders [24,35].

When asked about their preferred mode of sampling for the next round of cervical cancer screening, participants in this study demonstrated an almost equal distribution between self-sampling (42.6%) and clinician-collected cervical sampling (45,9%), with a smaller proportion reporting no clear preference (11.5%). This distribution aligns with findings from settings where women can choose their screening method, though the balance varies by programme and population. In England, when self-sampling was offered at invitation, 51.4% intended to choose it, 36.5% preferred clinician-based screening, and 10.5% were unsure [[36,37]]. In the Belgian VALHUDES study among screening participants, 57% preferred self-sampling for the next round and 41% preferred clinician collection [38]. Among colposcopy clinic attenders – more comparable to our secondary recruitment setting – preferences were more mixed: 37% preferred self-sampling, 35% had no preference, and smaller proportions favoured clinician-collected options [39]. Evidence from neighbouring Estonia, which has a similar cervical cancer burden to Latvia but more advanced HR-HPV-based screening, also supports these patterns. Estonian studies show high acceptability of HPV self-sampling and increased participation, especially among previous non-attenders, with few women preferring clinician-based sampling after trying self-sampling [12,13,40–43]. Overall, these comparisons indicate that Latvia’s near-equal split between clinician and self-sampling preferences is plausible during transition, and that indecision may be more common in clinically followed populations, underscoring the value of offering choice and clear information to build confidence in self-sampling.

Although this study was carried out in Latvia, its results may be applicable to other countries confronting comparable obstacles in cervical cancer prevention, especially those with a high disease burden, historically low screening uptake, or that are in the process of shifting from cytology-based to HR-HPV-based screening. Evidence from these settings suggests that emotional and psychosocial dimensions – such as perceived self-efficacy, autonomy, and reassurance about test accuracy – are central to shaping the acceptability of self-sampling, often exerting greater influence than practical aspects alone. Against this backdrop, the almost evenly split preferences observed in our study, together with the strong link between confidence and preference for self-sampling, highlight the need for implementation approaches that not only make self-sampling available but also explicitly foster confidence through clear instructions, education, and effective communication. These findings may thus guide HR-HPV self-sampling initiatives outside the Baltic region, particularly in contexts where building trust and ensuring long-term engagement remain major challenges.

In this study, most women reported no negative emotional/psychological responses during HR-HPV self-sampling. These findings suggest that self-sampling is generally perceived as emotionally acceptable and non-threatening. Emotional responses are important to consider, as they influence willingness to repeat the test and recommend it to others. Women’s feelings during the procedure, particularly comfort, confidence, and a sense of control, play a central role in building trust in self-sampling and promoting sustained participation in screening [44–46]. These results highlight that emotional acceptance, alongside technical reliability, should be prioritized when integrating HPV self-sampling into national screening programs.

The present study also explored participants’ emotional/psychological responses to HR-HPV self-sampling and how these feelings related to their preferred sampling method for the next round. Women who preferred clinician-collected cervical sampling in the next screening round more frequently reported embarrassment (6.4%) and discomfort (18.2%) during the procedure, whereas those who favored self-sampling in the next screening round predominantly described neutral or positive emotions (embarrassment 1.6%; discomfort 9.4%). These findings are consistent with international studies demonstrating that self-sampling reduces embarrassment and discomfort compared with clinician-based methods, likely because it enhances privacy, autonomy, and perceived control [24,39,44,45]. In our study, women who preferred self-sampling in the next screening round also expressed stronger confidence in performing the procedure (23.2% vs. 17.1%), suggesting increasing trust in their ability to collect samples correctly.

Importantly, our results indicate that positive emotional factors, especially confidence, may exert a stronger influence on preferences for screening modality than simply minimizing negative emotions such as embarrassment or discomfort. Although mitigating negative emotional reactions is likely a necessary step toward enhancing acceptability, it may not be enough to ensure ongoing use if women do not feel confident in their ability to carry out self-sampling correctly or to trust the test result. This view is consistent with prior research showing that worries about collecting the sample properly and doubts about result accuracy remain among the most frequently cited reasons for favoring clinician-collected sampling, even when self-sampling is regarded as less embarrassing [23–26].

While acceptability is multifaceted, the emotional dimension examined here provides valuable insight into mechanisms influencing screening preferences. From a behavioural standpoint, confidence can be viewed as an indicator of perceived self-efficacy and perceived behavioural control, both of which are central determinants of health-related actions [23–26]. In this framework, the self-sampling experience may shape emotional reactions, with confidence potentially mediating the relationship between that experience and subsequent screening preferences. While the cross-sectional nature of this study precludes causal conclusions, this proposed pathway offers a credible account of the observed relationships and highlights the need for confidence-enhancing strategies when implementing HR-HPV self-sampling within screening programmes.

Analysis involving multiple variables identified several important factors associated with women’s preference for clinician-collected cervical sampling. In our study, non-Latvian women were more likely either to prefer clinician-collected sampling or to report uncertainty regarding future screening modality. These findings may reflect cultural, linguistic, or informational differences influencing trust in self-sampling and confidence in performing the procedure independently. Previous studies have similarly shown that some ethnic minority groups may be less likely to engage with HR-HPV self-sampling because of concerns regarding test accuracy, unfamiliarity with the method, or preferences for direct interaction with healthcare professionals [30,47]. At the same time, recent evidence suggests that attitudes toward self-sampling among ethnically diverse populations are complex and context-dependent. A qualitative study from Northwest England reported that many women from ethnically diverse backgrounds considered self-sampling acceptable and empowering because of the increased privacy, convenience, and autonomy associated with the method, although concerns regarding correct sample collection and trust in test accuracy persisted among some participants [48]. Similarly, findings from the *YouScreen* study demonstrated that women who had previously not attended screening often reported positive experiences with self-sampling and expressed willingness to use it again in future screening rounds [35]. These findings suggest that cultural background alone does not determine acceptability; rather, access to understandable information, confidence in the procedure, health literacy, and trust in healthcare systems may play more important roles in shaping screening preferences.

An interesting finding was that educational level was not independently associated with screening modality preference in the adjusted analyses, suggesting that formal education alone may not adequately explain women’s screening preferences in this population. Instead, factors such as health literacy, trust in healthcare systems, perceived accuracy of self-sampling, and familiarity with screening procedures may play more important roles. Religious affiliation was not assessed in the present study; therefore, no direct conclusions regarding religious influences can be drawn. Nevertheless, cultural norms and attitudes toward intimate health procedures may influence the acceptability of HPV self-sampling in some populations. Together, these findings highlight the importance of culturally sensitive communication strategies, multilingual educational materials, and clear explanations regarding the accuracy and safety of HR-HPV self-sampling when implementing population-based screening programmes [48,35].

In our study, women who had not visited a gynecologist for more than three years were significantly less likely to prefer clinician-collected cervical sampling, and even those whose last visit occurred one to three years earlier showed a reduced preference compared to those women who had visited a gynecologist in a year. This suggests that self-sampling may appeal to women who are less regularly engaged with gynecological care. While this variable does not directly measure participation in cervical cancer screening, it may indirectly reflect patterns of healthcare utilization. Similar findings have been reported in other studies, where self-sampling was found to be particularly attractive to women who attend healthcare services less frequently [37,49–53].

Lifestyle-related factors also appeared to play a role. Although the mechanism is not entirely clear, smoking behavior may act as a proxy for broader lifestyle and health-related attitudes. Previous research has shown that smokers are generally less likely to participate in cervical cancer screening and other preventive health programs, possibly due to lower health awareness, reduced engagement with healthcare, and higher perceived barriers to screening participation [54,55]. Therefore, the observed association in our study may reflect differences in preventive health-seeking behavior and perceived convenience rather than smoking itself.

An interesting finding was a lower preference for self-sampling among women recruited from colposcopy settings. This finding is consistent with previous studies showing that women with recent or ongoing clinical follow-up because of changes in their cervix tend to prefer clinician-collected cervical sampling [37,56]. The reasons why these women opted for clinician-collected cervical sampling remain speculative, but there is a theory that respondents attending colposcopy are already engaged with screening and have a divergent perception of cervical cancer risk. This finding aligns with the review by Nelson et al., which reported that women who preferred clinician-collected sampling often expressed doubts about their ability to perform self-sampling correctly and about the reliability of the obtained specimens [24]. Nishimura et al. identified concerns among some women that self-sampling might reduce opportunities for direct consultation with healthcare professionals, leading them to favor clinician-collected sampling [25]. Therefore, lack of confidence in performing the procedure and the perceived importance of clinical interaction appear to be key factors influencing preference for clinician-collected cervical sampling [24,25,39].

Among participants who reported no clear preference regarding the screening modality (“hard to say”), a higher body mass index (BMI ≥ 25) was one independent predictor in the adjusted model. Women with higher BMI were approximately twice as likely to express indecision, which may reflect body image concerns, perceived procedural difficulty, or the trustworthiness of each modality. Also, other study data suggest that women with obesity are historically under-screened for cervical cancer, potentially due to physical discomfort with speculum exams, embarrassment, or stigma [57].

In addition, non-Latvian nationality was associated with a higher likelihood of expressing no preference, suggesting that cultural, linguistic, or informational barriers may influence screening-related decision-making [37,47,58,59]. Overall, emotional and experiential factors appeared more influential than standard sociodemographic characteristics in shaping preferences.

The study has several limitations. Behavioural data were self-reported and may be subject to reporting or recall bias; however, this approach is widely accepted in epidemiological research [60]. The general population sample was not randomly selected, which may limit representativeness, although geographical diversity was ensured. Additionally, self-sampling was performed in a healthcare setting rather than fully at home, which may have influenced emotional responses and limit generalisability to population-based programmes. Despite these limitations, the study provides valuable insight into women’s emotional responses and screening preferences within a real-world national context.

This study provides the first evidence from Latvia on women’s feelings during HR-HPV self-sampling and their preferences for future cervical cancer sampling modalities. The findings indicate that self-sampling is well tolerated and that reluctance is driven primarily by informational, emotional, and cultural factors rather than socioeconomic ones. Targeted communication strategies addressing confidence, accuracy, and follow-up—particularly for women of non-Latvian nationality and those with higher BMI—will be critical to ensure equitable implementation of HR-HPV self-sampling within national screening programmes.

## Conclusion

Most participants reported positive or neutral emotional/psychological responses during HR-HPV self-sampling, indicating that the procedure is emotionally well-tolerated. Embarrassment and discomfort were uncommon, while feelings of confidence and interest predominated. Self-sampling appeared particularly appealing to women less regularly engaged in gynecological care, suggesting its potential to reach under-screened populations. In contrast, preference for clinician-collected cervical sampling was more strongly influenced by medical history and cultural background. Importantly, confidence emerged as a key emotional factor associated with preference for self-sampling, suggesting that fostering trust in women’s ability to perform the procedure and in the reliability of test results is central to acceptance. These aspects should be taken into account when developing communication and education strategies for the introduction of HR-HPV self-sampling into the national screening program.

### Key points

1. Most women reported positive or neutral feelings during HR-HPV self-sampling, confirming it as an emotionally acceptable and well-tolerated procedure.
2. Preferences for self-sampling and clinician-collected cervical sampling were almost evenly distributed, indicating that both methods are widely acceptable among Latvian women.
3. Non-Latvian nationality and higher BMI were associated with greater uncertainty about preferred sampling methods, suggesting the need for tailored communication when introducing HPV self-sampling in Latvia’s screening program.

## Supporting information

S1 Table Sociodemographic, behavioral, and health-related characteristics of women by preferred mode of HR-HPV testing. (DOCX)
