# Supplementary material for: Factors associated with women’s choice for mode of cervical sampling in future cervical cancer screening
Source: PLoS One. 2026 Jul 9;21(7):e0353180. doi: 10.1371/journal.pone.0353180 (PMC13349179; doi:10.1371/journal.pone.0353180)
Supplement: S1 Table — (DOCX) [file pone.0353180.s001.docx]

**Supporting information**

**S1 table.** **Sociodemographic, behavioral, and health-related characteristics of women by preferred mode of HR-HPV testing.**

| **Variable** | **HR-HPV self-sampling** | | **Clinitian collected sampling** | | **Hard to say** | | **Total** | |
| --- | --- | --- | --- | --- | --- | --- | --- | --- |
|  | **n** | **%** | **n** | **%** | **n** | **%** | **n** | **%** |
| **Age (years)** | | | | | | | | |
| ≤49 | 401 | 77.3 | 436 | 78.1 | 102 | 72.9 | 939 | 77.2 |
| 50+ | 118 | 22.7 | 122 | 21.9 | 38 | 27.1 | 278 | 22.8 |
| **Nationality** | | | | | | | | |
| Non-Latvian | 112 | 21.6 | 152 | 27.2 | 42 | 30.0 | 306 | 25.1 |
| Latvian | 407 | 78.4 | 406 | 72.8 | 98 | 70.0 | 911 | 74.9 |
| **Marital status** | | | | | | | | |
| Divorced, widow | 62 | 11.9 | 74 | 13.3 | 21 | 15.0 | 157 | 12.9 |
| Married, cohabiting | 363 | 69.9 | 408 | 73.1 | 90 | 64.3 | 861 | 70.7 |
| Single | 94 | 18.1 | 76 | 13.6 | 29 | 20.7 | 199 | 16.4 |
| **Education** | | | | | | | | |
| Primary, secondary | 237 | 45.7 | 245 | 43.9 | 74 | 52.9 | 556 | 45.7 |
| University | 282 | 54.3 | 313 | 56.1 | 66 | 47.1 | 661 | 54.3 |
| **Women’s monthly income neto in last year (EUR)** | | | | | | | | |
| ≤400 | 114 | 22.0 | 119 | 21.3 | 33 | 23.6 | 266 | 21.9 |
| 401-1000 | 316 | 60.9 | 347 | 62.2 | 85 | 60.7 | 748 | 61.5 |
| 1001+ | 89 | 17.1 | 92 | 16.5 | 22 | 15.7 | 203 | 16.7 |
| **Smoking status** | | | | | | | | |
| Daily | 136 | 26.2 | 133 | 23.8 | 46 | 32.9 | 315 | 25.9 |
| Sometimes, quit | 133 | 25.6 | 120 | 21.5 | 29 | 20.7 | 282 | 23.2 |
| Never | 250 | 48.2 | 305 | 54.7 | 65 | 46.4 | 620 | 50.9 |
| **Alcohol use** | | | | | | | | |
| Once per week or more often | 35 | 7.1 | 30 | 5.7 | 12 | 9.0 | 77 | 6.7 |
| Rarely | 252 | 51.1 | 279 | 53.4 | 63 | 47.4 | 594 | 51.7 |
| Never | 206 | 41.8 | 213 | 40.8 | 58 | 43.6 | 477 | 41.6 |
| **Number of lifetime sex partners** | | | | | | | | |
| 4+ | 284 | 56.7 | 283 | 54.2 | 72 | 57.6 | 639 | 55.7 |
| 2-3 | 141 | 28.1 | 163 | 31.2 | 30 | 24.0 | 334 | 29.1 |
| 0-1 | 76 | 15.2 | 76 | 14.6 | 23 | 18.4 | 175 | 15.2 |
| **BMI** | | | | | | | | |
| 25+ | 227 | 44.3 | 250 | 45.1 | 84 | 60.9 | 561 | 46.6 |
| <25 | 285 | 55.7 | 304 | 54.9 | 54 | 39.1 | 643 | 53.4 |
| **Chronic diseases** | | | | | | | | |
| Yes | 176 | 33.9 | 189 | 33.9 | 51 | 36.4 | 416 | 34.2 |
| No | 343 | 66.1 | 369 | 66.1 | 89 | 63.6 | 801 | 65.8 |
| **Last visit to gynaecologist** | | | | | | | | |
| Don’t know, never | 29 | 5.6 | 42 | 7.5 | 12 | 8.6 | 83 | 6.8 |
| More than 3 years ago | 45 | 8.7 | 17 | 3.0 | 19 | 13.6 | 81 | 6.7 |
| 1-3 years ago | 131 | 25.2 | 110 | 19.7 | 25 | 17.9 | 266 | 21.9 |
| Last year | 314 | 60.5 | 389 | 69.7 | 84 | 60.0 | 787 | 64.7 |
| **Study group** | | | | | | | | |
| Colposcopy | 160 | 30.8 | 246 | 44.1 | 42 | 30.0 | 448 | 36.8 |
| General population | 359 | 69.2 | 312 | 55.9 | 98 | 70.0 | 769 | 63.2 |
